# Frequency of Breakfast, Lunch, and Dinner and Incidence of Proteinuria: A Retrospective Cohort Study

**DOI:** 10.3390/nu12113549

**Published:** 2020-11-19

**Authors:** Ryohei Tomi, Ryohei Yamamoto, Maki Shinzawa, Yoshiki Kimura, Yoshiyuki Fujii, Katsunori Aoki, Shingo Ozaki, Ryuichi Yoshimura, Manabu Taneike, Kaori Nakanishi, Makoto Nishida, Keiko Yamauchi-Takihara, Takashi Kudo, Yoshitaka Isaka, Toshiki Moriyama

**Affiliations:** 1 Department of Nephrology, Osaka University Graduate School of Medicine, 2–2-D11 Yamadaoka, Suita, Osaka 565-0871, Japan; rtomi@kid.med.osaka-u.ac.jp (R.T.); shinzawa@kid.med.osaka-u.ac.jp (M.S.); kimukimu_y2k@infoseek.jp (Y.K.); jan@v101.vaio.ne.jp (Y.F.); nekohue@yahoo.co.jp (K.A.); shingo.oz@kid.med.osaka-u.ac.jp (S.O.); ryoshimura@kid.med.osaka-u.ac.jp (R.Y.); isaka@kid.med.osaka-u.ac.jp (Y.I.); moriyama@wellness.hss.osaka-u.ac.jp (T.M.); 2 Health and Counseling Center, Osaka University, 1–17 Machikaneyamacho, Toyonaka, Osaka 560-0043, Japan; taneike@cardiology.med.osaka-u.ac.jp (M.T.); k-nakanishi@wellness.hss.osaka-u.ac.jp (K.N.); mnishida@wellness.hss.osaka-u.ac.jp (M.N.); takihara@wellness.hss.osaka-u.ac.jp (K.Y.-T.); kudo@psy.med.osaka-u.ac.jp (T.K.); 3 Health Promotion and Regulation, Department of Health Promotion Medicine, Osaka University Graduate School of Medicine, 1–17 Machikaneyamacho, Toyonaka, Osaka 560-0043, Japan

**Keywords:** proteinuria, breakfast, lunch, dinner, retrospective cohort study

## Abstract

Although multiple studies have revealed a close association of skipping breakfast with cardiometabolic diseases, few studies have reported its association with chronic kidney disease (CKD). Furthermore, there is scant reporting on the clinical impacts that skipping lunch and dinner has on cardiometabolic diseases and CKD. This retrospective cohort study, including 5439 female and 4674 male workers of a national university in Japan who underwent annual health checkups between January 2005 and March 2013, aimed to assess an association of frequencies of breakfast, lunch, and dinner with incidence of proteinuria (dipstick urinary protein ≥1+). The incidence of proteinuria was observed in 763 (14.0%) females and 617 (13.2%) males during the median 4.3 and 5.9 years of the observational period, respectively. In females, skipping breakfast as well as skipping dinner, but not lunch, were associated with the incidence of proteinuria (adjusted hazard ratios of breakfast frequency of “every day”, “sometimes”, and “rarely”: 1.00 (reference), 1.35 (1.09–1.66), and 1.54 (1.22–1.94), respectively; those of dinner frequency of “every day” and “≤sometimes”: 1.00 (reference) and 1.31 (1.00–1.72), respectively). However, no association was observed in male workers. Skipping breakfast and skipping dinner were identified as risk factors of proteinuria in females, but not in males.

## 1. Introduction

Chronic kidney disease (CKD), characterized by low glomerular filtration rate (GFR) and/or proteinuria [1], has been recognized as one of the major global public health problems [2]. Besides a decrease in GFR [3], multiple studies have identified proteinuria as a risk factor for end-stage kidney disease [4], cardiovascular disease [5], and mortality [6]. To establish a prevention program against CKD, modifiable lifestyle risk factors for CKD should be identified and effective interventions for these defined. Some of the lifestyle risk factors that could be used as promising targets for CKD prevention include obesity [7], smoking [8,9], alcohol consumption [10,11], sleep deprivation [12,13], and unhealthy diet [14].

Several studies have shown that skipping breakfast is a risk factor for several cardiometabolic diseases, including metabolic syndrome [15], type 2 diabetes (T2D) [16], cardiovascular diseases (CVDs) [17], and all-cause mortality [17]. However, little information is available on the clinical impacts of skipping breakfast on CKD. A few cross-sectional studies reported high prevalence of proteinuria in adults who skipped breakfast [18,19]. Contrary to skipping breakfast, the clinical impacts of skipping lunch and dinner on cardiometabolic diseases have been scarcely investigated. The Health Professionals Follow-Up study reported that the number of meals consumed by an individual was associated with the incidence of T2D. This suggested that skipping lunch and/or dinner could contribute to the incidence of cardiometabolic diseases [20]. A cross-sectional study comprising 4370 Korean adults suggested that the subjects who reported a meal frequency <15 times/week exhibited a higher prevalence rate of microalbuminuria [19].

The aim of the present retrospective cohort study was to assess the association between the frequency of breakfast, lunch, and dinner and the incidence of proteinuria in 10,113 workers from a national university in Japan.

## 2. Materials and Methods

### 2.1. Study Population

**Figure 1:**
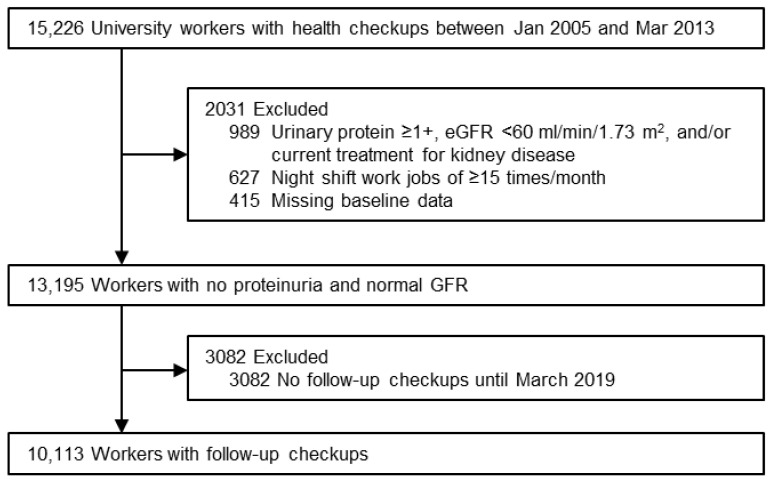
Flow diagram of the entry of participants.

The participants eligible for inclusion in the present retrospective cohort study were 15,226 workers, aged 19–60 years, from Osaka University. These individuals had visited the Health Care Center, Osaka University, for their annual health checkups during the entry period between January 2005 and March 2013. Of 14,237 (93.5%) workers with estimated GFR (eGFR) ≥ 60 mL/min/1.73 m^2^, a negative or trace result of urinary protein by dipstick test, and no current treatment for self-reported kidney disease at their first visit during the entry period, we excluded 627 (4.1%) workers with ≥15 night shift work jobs/month, 415 (2.7%) workers with missing baseline data at their first visit, and 3082 (20.2%) workers without a follow-up visit during the observational period between January 2005 and March 2019 (Figure 1). To calculate eGFR, the following Japanese equation was used: eGFR (mL/min/1.73 m^2^) = 194 × age (years)^−0.287^ × serum creatinine (mg/dL)^−1.094^ × 0.739 if female [21]. Finally, the present study included 10,113 (66.4%) workers with no proteinuria (dipstick urinary protein ≤±) and normal renal function (eGFR ≥ 60 mL/min/1.73 m^2^). All data were retrieved from the electronic database at the Health Care Center, Osaka University. The protocol of the present study was approved by the ethics committees of the Health and Counseling Center, Osaka University (No. 2020-8) and Osaka University Hospital (No. 17009-2). The present study used an opt-out approach to informed consent, according to Japanese Ethical Guidelines for Medical and Health Research Involving Human Subjects.

### 2.2. Measurements

The baseline demographic and clinical characteristics noted were age, sex, body mass index (BMI) calculated as weight in kilogram divided by the square of height in meters, systolic and diastolic blood pressure, and the levels of total cholesterol, triglyceride, hemoglobin A1c, and serum creatinine as well as the amount of protein in the random void urine sample, assessed using the dipstick method. The results of the urinary protein level were interpreted by well-trained nurses and recorded as negative (-), ±, 1+, 2+, 3+, or 4+.

The baseline lifestyle characteristics and current medical treatment were assessed with the use of self-administered questionnaires at the time of the baseline visit. The main exposure of the present study was the frequency of intake of breakfast, lunch, and dinner through the following three possible choices: “every day”, “sometimes”, and “rarely”. Since the number of individuals who responded with the option “rarely” in the context of their lunch and dinner frequencies was low (lunch, *n* = 21 (0.4%) and 67 (1.4%) in females and males, respectively; dinner, *n* = 9 (0.2%) and 4 (0.1%), respectively), we categorized the workers into two groups: subjects who responded with “every day” and subjects who responded with “≤sometimes.” As regards smoking status, the categories were non-smoker, past smoker, and current smoker, and the results were based on the response to a question of smoking with the following three possible responses: “I do not smoke”, “I quit smoking”, or “I smoke”. The frequency of alcohol consumption was ascertained with a specific query: “How often do you drink per week?” This question, too, had to be answered with the present responses of “rarely”, “1–3 days”, “4–6 days”, or “every day”. The frequency of indulging in snacks was also looked at, with the question “How often do you snack between meals?” and with the answer of “rarely”, “1 or 2 days”, “3–5 days”, or “≥6 days per week”. Diagnoses of hypertension, diabetes, dyslipidemia, and heart disease were made according to positive answers to the questions of current treatments for hypertension, diabetes, hyperlipidemia, and heart disease.

The outcome measure of the present study was the incidence of proteinuria, defined as urinary protein ≥1+ as assessed by the dipstick test. The observational period was designated as the time from the baseline visit to (i) the incidence of proteinuria or (ii) the last measurement of urinary protein before the end of March 2019, whichever came first.

### 2.3. Statistical Analysis

Baseline characteristics between each frequency of meal intakes were compared using the χ^2^ test, the *t*-test, one-way analysis of variance, the Kruskal–Wallis test, or the Wilcoxon rank sum test, as appropriate. To evaluate the association between the frequency of meals and incidence of proteinuria, the cumulative probabilities of the incidence of proteinuria were calculated using the Kaplan–Meier method and compared using the log-rank test and the Cox proportional hazards models. Model 1 included age (year), smoking status (non-, past, and current smokers), frequency of alcohol consumption (rarely, 1–3 days, 4–6 days, or every day per week), BMI (kg/m^2^), snacking frequency (rarely, 1 or 2 days, 3–5 days, or ≥6 days per week), systolic blood pressure (mmHg), total cholesterol (mg/dL), triglyceride (mg/dL), HbA1c (%), eGFR (mL/min/1.73 m^2^), urinary protein (- vs. ±), and current treatments for hypertension, dyslipidemia, diabetes mellitus, and cardiovascular disease (yes vs. no) as covariates. Model 2 added the frequency of intake of breakfast, lunch, and dinner as covariates. The proportional hazards assumption was checked using Schoenfeld residuals. All these analyses were performed in males and females, separately.

Continuous variables were expressed as mean ± standard deviation or median and interquartile range, as appropriate. The categorical variables were expressed as number and proportion. Statistical significance was set at *p* < 0.05. Statistical analyses were performed using Stata, version 15.0 (Stata Corp, www.stata.com).

## 3. Results

### 3.1. Baseline Characteristics

The baseline characteristics of 5439 female and 4674 male workers with a median age of 31 years (interquartile range, 26–38) and 34 years (29–42), respectively, are listed in Table 1 and Table 2. As observed, in both females and males, workers with a lower meal frequency were of younger age, were current smokers, and displayed higher values of body mass index, systolic blood pressure, total cholesterol, and hemoglobin A1c (Table 1). While the eGFR was comparable between both male and female workers, female workers with lower meal frequency consumed alcohol more frequently.

### 3.2. Skipping Meals and Risks for Proteinuria

**Figure 2:**
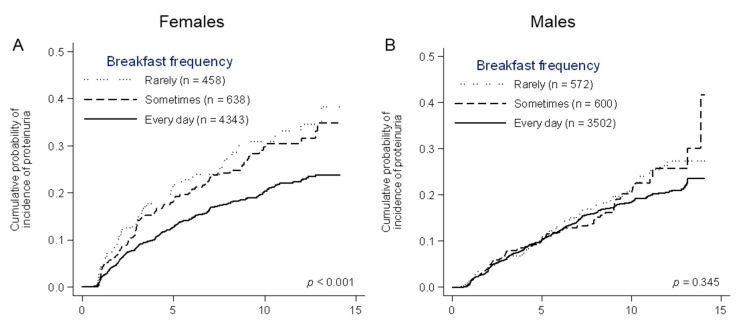

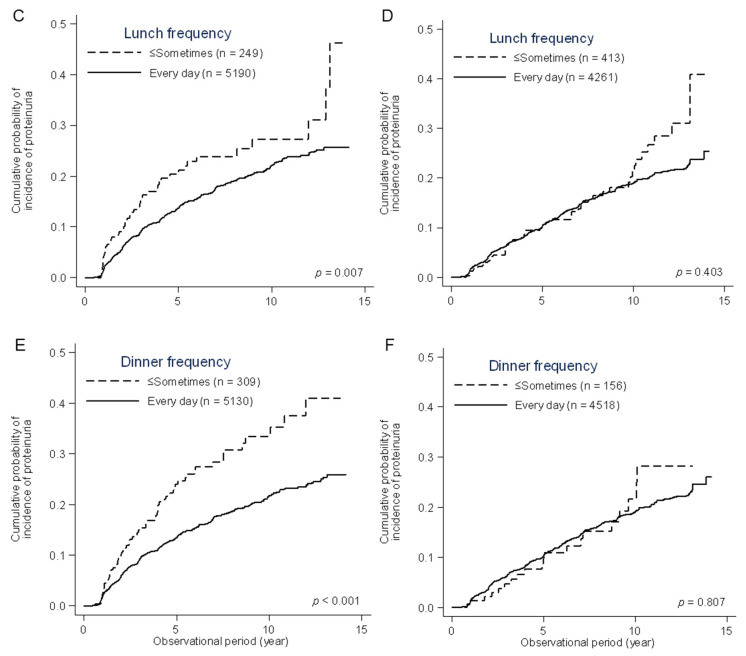
Cumulative probability of the incidence of proteinuria in females and males, stratified by the frequency of breakfast, lunch, and dinner. A significant difference was observed in the frequency of breakfast (**A**), lunch (**C**), and dinner (**E**) in females, not in males (**B**,**D**,**F**).

**Figure 3:**
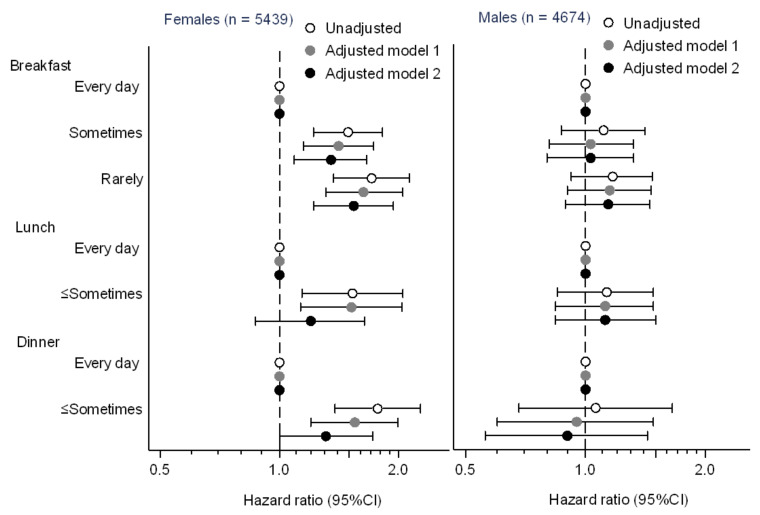
Associations of the frequency of breakfast, lunch, and dinner with the incidence of proteinuria. CI, confidence interval. Model 1 adjusted for age (year), smoking status (non-, past, and current smokers), drinking frequency (rarely, 1–3, 4–6, and 7 days/week), body mass index (kg/m^2^), snacking (rarely, 1 or 2, 3–5, and ≥6 days/week), systolic blood pressure (mg/dL), total cholesterol (mg/dL), triglyceride (mg/dL), HbA1c (%), estimated glomerular filtration rate (mL/min/1.73 m^2^), urinary protein (- and ±), and current treatments for hypertension, dyslipidemia, diabetes mellitus, and cardiovascular disease (yes vs. no). Model 2 adjusted for covariates in model 1 and the frequencies of breakfast, lunch, and dinner.

During the median 4.3 (interquartile range 2.0–7.7) years of the observational period, 763 (14.0%) females developed proteinuria (Table 3). A higher incidence rate of proteinuria was seen in female workers who skipped meals. With respect to frequency of breakfast intake, the incidence rates of proteinuria were 24.3 (95% confidence interval, 22.4–26.4), 35.8 (29.8–43.0), and 40.8 (33.2–50.0) per 1000 person-years in the “every day”, “sometimes”, and “rarely” groups, respectively. Skipping lunch was associated with the incidence of proteinuria as well (26.3 (4.5–28.3) and 40.0 (30.1–53.1) per 1000 person-years in the “every day” and “≤sometimes” group, respectively). A similar association was observed in skipping dinner (25.8 (24.0–27.8) and 46.3 (36.6–58.7) per 1000 person-years in “every day” and “≤sometimes” group, respectively). The cumulative incidence of proteinuria was significantly higher in the female workers who skipped breakfast, lunch, and dinner (*p* < 0.001, 0.007, and <0.001, respectively) (Figure 2A,C,E). Unadjusted Cox proportional hazards models showed that skipping breakfast, lunch, and dinner were significantly associated with the incidence of proteinuria (hazard ratios (95% confidence interval) for each category of breakfast frequency: every day 1.00 (reference), sometimes 1.49 (1.22–1.82), and rarely 1.71 (1.37–2.13), lunch frequency: every day 1.00 (reference) and ≤sometimes 1.53 (1.14–2.05), and dinner frequency: everyday 1.00 (reference) and ≤sometimes 1.77 (1.38–2.27)) (Table 3 and Figure 3). After adjusting for clinically relevant factors, skipping breakfast, lunch, and dinner were identified as significant predictors of the incidence of proteinuria (breakfast frequency: sometimes 1.41 (1.15–1.73) and rarely 1.63 (1.31–2.05); lunch frequency: ≤sometimes 1.52 (1.13–2.04); dinner frequency: ≤sometimes 1.55 (1.20–1.99)) (Adjusted model 1 in Table 3 and Figure 3). However, after adjusting for meal frequency, the association between lunch frequency and the incidence of proteinuria was remarkably attenuated (≤sometimes 1.20 (0.87–1.64)), whereas breakfast and dinner frequency still exhibited significant association with the incidence of proteinuria (breakfast frequency: sometimes 1.35 (1.09–1.66) and rarely 1.54 (1.22–1.94); dinner frequency: ≤sometimes 1.31 (1.00–1.72)) (Adjusted model 2 in Table 3 and Figure 3).

The incidence of proteinuria was observed in 617 (13.2%) male workers during the observational period of a median 5.9 years (2.5–9.5). In contrast to the female workers, the incidence of proteinuria was comparable among male workers with different meal frequencies (Table 3). Cumulative probabilities of the incidence of proteinuria were not significantly different according to meal frequencies (*p* = 0.345, 0.403, and 0.807 for breakfast, lunch, and dinner frequency, respectively) (Figure 2B,D,F). In Cox proportional hazards models, breakfast, lunch, and dinner frequencies were not associated with the incidence of proteinuria (Table 3 and Figure 3).

## 4. Discussion

The present retrospective cohort study identified skipping breakfast and dinner as significant predictors of the incidence of proteinuria in female workers, whereas the same association could not be established in male workers. Upon confirmation of the clinical relevance of skipping breakfast with effects on the kidney, the present study identified that skipping dinner had a potentially deleterious effect on the kidney. Several advantages of the present study included the large sample size (*n* = 10,113) and the long observational period (the median period of 4.3 and 5.9 years in female and male workers, respectively). These enabled a statistically meaningful analysis of the clinical impact of skipping dinner even in the presence of a low prevalence rate of 4.6%.

Earlier, a few cross-sectional studies reported an association between the meal frequency and the prevalence of proteinuria. A large cross-sectional study, including 60,800 Japanese adults aged 20–75 years, reported a significant association between skipping breakfast and the high prevalence of proteinuria in both males and females [18]. Another cross-sectional study including 4370 Korean adults showed that skipping breakfast was associated with high prevalence of proteinuria, defined as urinary albumin/creatinine ratio of ≥30 mg/g in males, but not in females [19]. In this Korean study, the frequency of lunch and dinner intake was not found to be associated with the prevalence of proteinuria. The present study identified avoidance of breakfast and dinner as key predictors of proteinuria especially in females. Since skipping breakfast and dinner leads to a longer fasting time than skipping lunch, the results of the present study suggest that a longer fasting time may affect the incidence of proteinuria.

Although the precise mechanism for the association between longer fasting time and the incidence of proteinuria is unknown, one of the potential explanations could be the Staub–Traugott (or second meal) effect, which states that skipping a meal induces an increase in postprandial hyperglycemia and impaired insulin response in a subsequent meal [22]. Several randomized controlled studies have demonstrated that, upon skipping breakfast, postprandial hyperglycemia was observed after subsequent lunch and even dinner [23,24]. Postprandial hyperglycemia induces oxidative stress and vascular endothelial dysfunction [25] and is regarded as a risk factor for cardiovascular diseases [26,27]. Furthermore, it was reported earlier that, because of the Staub–Traugott effect, people who skip breakfast showed higher levels of oxidative stress [28] and vascular endothelial dysfunction [29], possibly leading to the incidence of proteinuria [30].

While the basic reason behind the difference in the results with respect to sex cannot be ascertained, one of the potential contributors to this difference may be dysmenorrhea. Several cross-sectional studies reported that skipping breakfast was associated with a higher prevalence of dysmenorrhea in young females [31,32] and that young females with dysmenorrhea have higher levels of proinflammatory cytokines [33] and oxidative stress [33,34], compared with those without dysmenorrhea. Since proinflammatory cytokines and oxidative stress are major risk factors of proteinuria [30], females who skip breakfast may be at higher risk of developing proteinuria than those who have breakfast regularly. Another potential contributor to the observed sex-specific difference in results could be a difference in lifestyles, including low consumption of vegetables [35] and fish [36], which are associated with the incidence of proteinuria. As previous cross-sectional studies reported that skipping breakfast was associated with lower consumption of vegetable [37] and fish [38] in females, but not in males, females who skipped breakfast might be at higher risk of proteinuria than those who did not. Further studies are essential to clarify the sex-dependent association between skipping breakfast and the incidence of proteinuria.

The present study has several limitations. First, the meal frequency was categorized into only three groups: every day, sometimes, and rarely. The minimum frequency of breakfast and dinner, which had a minor influence on the incidence of proteinuria, was not identified in the present study. More information is essential to assess the optimal meal frequency. Second, the dose-dependent association between the frequency of lunch and dinner could not be assessed because of the very low prevalence of males and females who eat lunch and dinner rarely (*n* = 21 and 9 in females, respectively, and 67 and 4 in males, respectively). To assess their dose-dependent associations, a larger cohort is necessary. Third, the present study included mainly young workers from one of the largest national universities in Japan. This group mainly consisted of office workers, academic researchers, and health care workers at university hospitals. The generalizability of the results of the present study should also be verified in different cohorts, especially in an elderly population. The Korean cross-sectional study reported that skipping breakfast was associated more strongly with the prevalence of proteinuria in the older adults aged >42 years, when compared with the younger adults aged <42 years, suggesting an age-dependent association between skipping breakfast and the prevalence of proteinuria [19]. Fourth, dietary patterns, including the Dietary Approaches to Stop Hypertension (DASH) and Mediterranean dietary patterns, were not available in the present study. Because previous studies suggested that these dietary patterns might be useful in preventing CKD [39], the results of the present study were confounded by these dietary patterns. Further studies are essential to assess an association among meal frequency, dietary patterns, and the incidence of proteinuria.

## 5. Conclusions

The present retrospective cohort study of 10,113 university workers showed that skipping breakfast and dinner were risk factors for proteinuria in females, but not in males. Furthermore, the results indicated that skipping meals may be one of the potentially modifiable lifestyles for the prevention of CKD, especially in female workers.

## Figures and Tables

**Table 1:** Baseline characteristics stratified by the frequency of breakfast, lunch, and dinner in 5439 females.

	Breakfast			Lunch		Dinner	
	Every Day	Sometimes	Rarely	Every Day	≤Sometimes	Every Day	≤Sometimes
Number	4343	638	458	5190	249	5130	309
Age (years) *^,‡^	32 (26–39)	29 (25–34)	29 (26–34)	31 (26–38)	30 (26–37)	31 (26–39)	29 (25–34)
Non-smokers, *n* (%) *^,†,‡^	3990 (91.9)	559 (87.6)	368 (80.3)	4699 (90.5)	218 (87.6)	4659 (90.8)	258 (83.5)
Past smokers	195 (4.5)	39 (6.1)	36 (7.9)	260 (5.0)	10 (4.0)	250 (4.9)	20 (6.5)
Current smokers	158 (3.6)	40 (6.3)	54 (11.8)	231 (4.5)	21 (8.4)	221 (4.3)	31 (10.0)
Drinking, Rarely, *n* (%) *	2794 (64.3)	371 (58.2)	289 (63.1)	3308 (63.7)	146 (58.6)	3274 (63.8)	180 (58.3)
1–3 days/week	1111 (25.6)	192 (30.1)	107 (23.4)	1339 (25.8)	71 (28.5)	1324 (25.8)	86 (27.8)
4–6	190 (4.4)	37 (5.8)	27 (5.9)	243 (4.7)	11 (4.4)	237 (4.6)	17 (5.5)
7	248 (5.7)	38 (6.0)	35 (7.6)	300 (5.8)	21 (8.4)	295 (5.8)	26 (8.4)
Snacking, Rarely, *n* (%) ^†,‡^	557 (12.8)	85 (13.3)	73 (15.9)	666 (12.8)	49 (19.7)	656 (12.8)	59 (19.1)
1 or 2 days/week	1322 (30.4)	190 (29.8)	127 (27.7)	1563 (30.1)	76 (30.5)	1534 (29.9)	105 (34.0)
3–5	1068 (24.6)	174 (27.3)	103 (22.5)	1288 (24.8)	57 (22.9)	1276 (24.9)	69 (22.3)
≥6	1396 (32.1)	189 (29.6)	155 (33.8)	1673 (32.2)	67 (26.9)	1664 (32.4)	76 (24.6)
Body mass index, kg/m^2^	20.6 ± 2.8	20.7 ± 2.8	20.4 ± 2.9	20.6 ± 2.8	20.6 ± 2.9	20.6 ± 2.8	20.9 ± 3.3
Systolic BP, mmHg	107 ± 12	106 ± 12	107 ± 12	107 ± 12	107 ± 13	107 ± 12	106 ± 11
Diastolic BP, mmHg	66 ± 10	65 ± 10	65 ± 10	66 ± 10	65 ± 10	66 ± 10	64 ± 9
Total cholesterol, mg/dL ^‡^	183 (165–205)	183 (164–204)	187 (168–205)	183 (165–205)	184 (164–202)	184 (165–205)	179 (162–199)
Triglyceride, mg/dL ^†,‡^	51 (39–68)	50 (39–70)	50 (39–69)	51 (39–68)	48 (36–67)	51 (39–68)	48 (37–66)
Hemoglobin A1c, % *	5.3 ± 0.3	5.2 ± 0.3	5.2 ± 0.3	5.2 ± 0.3	5.2 ± 0.3	5.2 ± 0.3	5.2 ± 0.3
eGFR, ml/min/1.73 m^2^ *	93 (82–103)	96 (86–107)	97 (86–108)	93 (83–104)	92 (84–106)	93 (83–104)	94 (85–108)
Urinary protein (-), *n* (%) ^‡^	3946 (90.9)	582 (91.2)	415 (90.6)	4720 (90.9)	223 (89.6)	4676 (91.2)	267 (86.4)
(±)	397 (9.1)	56 (8.8)	43 (9.4)	470 (9.1)	26 (10.4)	454 (8.8)	42 (13.6)
Current treatments for							
Hypertension, *n* (%)	36 (0.8)	4 (0.6)	2 (0.4)	39 (0.8)	3 (1.2)	42 (0.8)	0 (0.0)
Dyslipidemia, *n* (%)	24 (0.6)	2 (0.3)	1 (0.2)	25 (0.5)	2 (0.8)	26 (0.5)	1 (0.3)
Diabetes mellitus, *n* (%)	5 (0.1)	0 (0.0)	0 (0.0)	5 (0.1)	0 (0.0)	5 (0.1)	0 (0.0)
CVD, *n* (%)	6 (0.1)	0 (0.0)	0 (0.0)	6 (0.1)	0 (0.0)	6 (0.1)	0 (0.0)
Breakfast, Every day, *n* (%) ^†,‡^				4238 (81.7)	105 (42.2)	4199 (81.9)	144 (46.6)
Sometimes				565 (10.9)	73 (29.3)	544 (10.6)	94 (30.4)
Rarely				387 (7.5)	71 (28.5)	387 (7.5)	71 (23.0)
Lunch Every day, *n* (%) *^,‡^	4238 (97.6)	565 (88.6)	387 (84.5)			4974 (97.0)	216 (69.9)
≤Sometimes	105 (2.4)	73 (11.4)	71 (15.5)			156 (3.0)	93 (30.1)
Dinner Every day, *n* (%) *^,†^	4199 (96.7)	544 (85.3)	387 (84.5)	4974 (95.8)	156 (62.7)		
≤Sometimes	144 (3.3)	94 (14.7)	71 (15.5)	216 (4.2)	93 (37.3)		

Mean ± standard deviation; median (25–75%); SBP, systolic blood pressure; DBP, diastolic blood pressure; eGFR, estimated glomerular filtration rate; CVD, cardiovascular disease; * *p* < 0.05 among 3 categories of breakfast frequency; † *p* < 0.05 between 2 categories of lunch frequency; ‡ *p* < 0.05 between 2 categories of dinner frequency.

**Table 2:** Baseline characteristics stratified by the frequency of breakfast, lunch, and dinner in 4674 males.

	Breakfast			Lunch		Dinner	
	Every Day	Sometimes	Rarely	Every Day	≤Sometimes	Every Day	≤Sometimes
Number	3502	600	572	4261	413	4518	156
Age (years) *^,†,‡^	35 (30–44)	32 (28–37)	32 (28–37)	34 (30–42)	32 (29–38)	34 (30–42)	32 (28–38)
Non-smokers, *n* (%) *^,†,‡^	2645 (75.5)	387 (64.5)	331 (57.9)	3091 (72.5)	272 (65.9)	3269 (72.4)	94 (60.3)
Past smokers	399 (11.4)	70 (11.7)	67 (11.7)	497 (11.7)	39 (9.4)	522 (11.6)	14 (9.0)
Current smokers	458 (13.1)	143 (23.8)	174 (30.4)	673 (15.8)	102 (24.7)	727 (16.1)	48 (30.8)
Drinking, Rarely, *n* (%)	1498 (42.8)	245 (40.8)	230 (40.2)	1794 (42.1)	179 (43.3)	1908 (42.2)	65 (41.7)
1–3 days/week	1035 (29.6)	195 (32.5)	176 (30.8)	289 (30.3)	117 (28.3)	1357 (30.0)	49 (31.4)
4–6	393 (11.2)	71 (11.8)	61 (10.7)	471 (11.1)	54 (13.1)	505 (11.2)	20 (12.8)
7	576 (16.4)	89 (14.8)	105 (18.4)	707 (16.6)	63 (15.3)	748 (16.6)	22 (14.1)
Snacking, Rarely, *n* (%) *	1486 (42.4)	249 (41.5)	293 (51.2)	1827 (42.9)	201 (48.7)	1963 (43.4)	65 (41.7)
1 or 2 days/week	1193 (34.1)	211 (35.2)	146 (25.5)	1421 (33.3)	129 (31.2)	1500 (33.2)	50 (32.1)
3–5	473 (13.5)	94 (15.7)	69 (12.1)	590 (13.8)	46 (11.1)	614 (13.6)	22 (14.1)
≥6	350 (10.0)	46 (7.7)	64 (11.2)	423 (9.9)	37 (9.0)	441 (9.8)	19 (12.2)
Body mass index, kg/m^2^ *	23.2 ± 3.1	23.3 ± 3.5	22.8 ± 3.1	23.1 ± 3.2	23.1 ± 3.3	23.1 ± 3.2	23.3 ± 3.5
Systolic BP, mmHg ^‡^	118 ± 14	118 ± 13	117 ± 14	118 ± 14	117 ± 13	118 ± 14	115 ± 13
Diastolic BP, mmHg ^‡^	75 ± 12	75 ± 12	74 ± 12	75 ± 12	75 ± 12	75 ± 12	73 ± 12
Total cholesterol, mg/dL	192 (171–215)	192 (172–214)	191 (171–216)	192 (171–215)	189 (169–213)	192 (171–215)	189 (167–214)
Triglyceride, mg/dL ^†^	82 (58–123)	80 (58–129)	78 (53–119)	83 (58–124)	73 (52–112)	81 (57–123)	86 (56–119)
Hemoglobin A1c, % *^,†,‡^	5.3 ± 0.5	5.2 ± 0.4	5.2 ± 0.4	5.3 ± 0.5	5.2 ± 0.4	5.3 ± 0.5	5.2 ± 0.3
eGFR, ml/min/1.73 m^2^ *^,†,‡^	86 (78–96)	89 (80–99)	90 (82–99)	86 (78–96)	91 (82–100)	87 (79–96)	91 (80–100)
Urinary protein (-), *n* (%) *^,‡^	3277 (93.6)	542 (90.3)	537 (93.9)	3971 (93.2)	385 (93.2)	4218 (93.4)	138 (88.5)
(±)	225 (6.4)	58 (9.7)	35 (6.1)	290 (6.8)	28 (6.8)	300 (6.6)	18 (11.5)
Hypertension, *n* (%)	103 (2.9)	16 (2.7)	7 (1.2)	119 (2.8)	7 (1.7)	124 (2.7)	2 (1.3)
Dyslipidemia, *n* (%) *	56 (1.6)	5 (0.8)	1 (0.2)	59 (1.4)	3 (0.7)	61 (1.4)	1 (0.6)
Diabetes mellitus, *n* (%) *	34 (1.0)	3 (0.5)	0 (0.0)	36 (0.8)	1 (0.2)	36 (0.8)	1 (0.6)
CVD, *n* (%)	9 (0.3)	1 (0.2)	1 (0.2)	10 (0.2)	1 (0.2)	11 (0.2)	0 (0.0)
Breakfast, Every day, *n* (%) ^†,‡^				3283 (77.0)	219 (53.0)	3424 (75.8)	78 (50.0)
Sometimes				498 (11.7)	102 (24.7)	560 (12.4)	40 (25.6)
Rarely				480 (11.3)	92 (22.3)	534 (11.8)	38 (24.4)
Lunch, Every day, *n* (%) *^,‡^	3283 (93.7)	498 (83.0)	480 (83.9)			4182 (92.6)	79 (50.6)
≤Sometimes	219 (6.3)	102 (17.0)	92 (16.1)			336 (7.4)	77 (49.4)
Dinner, Every day, *n* (%) *^,†^	3424 (97.8)	560 (93.3)	534 (93.4)	4182 (98.1)	336 (81.4)		
≤Sometimes	78 (2.2)	40 (6.7)	38 (6.6)	79 (1.9)	77 (18.6)		

Mean ± standard deviation; median (25–75%); SBP, systolic blood pressure; DBP, diastolic blood pressure; eGFR, estimated glomerular filtration rate; CVD, cardiovascular disease; * *p* < 0.05 among 3 categories of breakfast frequency; † *p* < 0.05 between 2 categories of lunch frequency; ‡ *p* < 0.05 between 2 categories of dinner frequency.

**Table 3:** Meal frequency and the incidence of proteinuria in 5439 females and 4674 males.

	Breakfast			Lunch		Dinner	
	Every Day	Sometimes	Rarely	Every Day	≤Sometimes	Every Day	≤Sometimes
Females							
Incidence of proteinuria, *n* (%)	557 (12.8)	114 (17.9)	92 (20.1)	715 (13.8)	48 (19.3)	694 (13.5)	69 (22.3)
IR per 1000 PY (95% CI)	24.3 (22.4–26.4)	35.8 (29.8–43.0)	40.8 (33.2–50.0)	26.3 (24.5–28.3)	40.0 (30.1–53.1)	25.8 (24.0–27.8)	46.3 (36.6–58.7)
Hazard ratio (95% CI)							
Unadjusted model	1.00 (reference)	1.49 (1.22–1.82)	1.71 (1.37–2.13)	1.00 (reference)	1.53 (1.14–2.05)	1.00 (reference)	1.77 (1.38–2.27)
Adjusted model 1	1.00 (reference)	1.41 (1.15–1.73)	1.63 (1.31–2.05)	1.00 (reference)	1.52 (1.13–2.04)	1.00 (reference)	1.55 (1.20–1.99)
Adjusted model 2	1.00 (reference)	1.35 (1.09–1.66)	1.54 (1.22–1.94)	1.00 (reference)	1.20 (0.87–1.64)	1.00 (reference)	1.31 (1.00–1.72)
Males							
Incidence of proteinuria, *n* (%)	454 (13.0)	79 (13.2)	84 (14.7)	562 (13.2)	55 (13.3)	597 (13.2)	20 (12.8)
IR per 1000 PY (95% CI)	20.6 (18.7–22.5)	22.7 (18.2–28.4)	23.9 (19.3–29.6)	21.0 (19.3–22.8)	23.7 (18.2–30.8)	21.2 (19.6–23.0)	22.3 (14.4–34.5)
Hazard ratio (95% CI)							
Unadjusted model	1.00 (reference)	1.11 (0.87–1.41)	1.17 (0.92–1.47)	1.00 (reference)	1.13 (0.85–1.48)	1.00 (reference)	1.06 (0.68–1.65)
Adjusted model 1	1.00 (reference)	1.03 (0.81–1.32)	1.15 (0.90–1.46)	1.00 (reference)	1.12 (0.84–1.48)	1.00 (reference)	0.95 (0.60–1.48)
Adjusted model 2	1.00 (reference)	1.03 (0.80–1.32)	1.14 (0.89–1.45)	1.00 (reference)	1.12 (0.84–1.50)	1.00 (reference)	0.90 (0.56–1.43)

IR, incidence rate; PY, person-years; CI, confidence interval; Model 1 adjusted for age (year), smoking status (non-, past, and current smokers), drinking frequency (rarely, 1–3, 4–6, and 7 days/week), body mass index (kg/m^2^), snacking; (rarely, 1 or 2, 3–5, and ≥6 days/week), systolic blood pressure (mmHg), total cholesterol (mg/dL), triglyceride (mg/dL), HbA1c (%), estimated glomerular filtration rate; (mL/min/1.73 m^2^), urinary protein (- and ±), and current treatments for hypertension, dyslipidemia, diabetes mellitus, and cardiovascular disease; Model 2 adjusted for covariates in model 1 and the frequencies of breakfast, lunch, and dinner.

## References

[1] Japanese Society of Nephrology (2019). Essential points from Evidence-based Clinical Practice Guidelines for Chronic Kidney Disease 2018. Clin. Exp. Nephrol..

[2] GBD Chronic Kidney Disease Collaboration (2020). Global, regional, and national burden of chronic kidney disease, 1990–2017: A systematic analysis for the Global Burden of Disease Study 2017. Lancet.

[3] Kanda E., Kashihara N., Matsushita K., Usui T., Okada H., Iseki K., Mikami K., Tanaka T., Wada T., Watada H. (2018). Guidelines for clinical evaluation of chronic kidney disease: AMED research on regulatory science of pharmaceuticals and medical devices. Clin. Exp. Nephrol..

[4] Coresh J., Heerspink H.J.L., Sang Y., Matsushita K., Arnlov J., Astor B.C., Black C., Brunskill N.J., Carrero J.J., Feldman H.I. (2019). Change in albuminuria and subsequent risk of end-stage kidney disease: An individual participant-level consortium meta-analysis of observational studies. Lancet Diabetes Endocrinol..

[5] Nagai K., Yamagata K., Iseki K., Moriyama T., Tsuruya K., Fujimoto S., Narita I., Konta T., Kondo M., Kasahara M. (2019). Cause-specific mortality in the general population with transient dipstick-proteinuria. PLoS ONE.

[6] Iseki K., Konta T., Asahi K., Yamagata K., Fujimoto S., Tsuruya K., Narita I., Kasahara M., Shibagaki Y., Moriyama T. (2018). Dipstick proteinuria and all-cause mortality among the general population. Clin. Exp. Nephrol..

[7] Garofalo C., Borrelli S., Minutolo R., Chiodini P., De Nicola L., Conte G. (2017). A systematic review and meta-analysis suggests obesity predicts onset of chronic kidney disease in the general population. Kidney Int..

[8] Xia J., Wang L., Ma Z., Zhong L., Wang Y., Gao Y., He L., Su X. (2017). Cigarette smoking and chronic kidney disease in the general population: A systematic review and meta-analysis of prospective cohort studies. Nephrol. Dial. Transplant..

[9] Ito K., Maeda T., Tada K., Takahashi K., Yasuno T., Masutani K., Mukoubara S., Arima H., Nakashima H. (2020). The role of cigarette smoking on new-onset of chronic kidney disease in a Japanese population without prior chronic kidney disease: Iki epidemiological study of atherosclerosis and chronic kidney disease (ISSA-CKD). Clin. Exp. Nephrol..

[10] Kimura Y., Yamamoto R., Shinzawa M., Isaka Y., Iseki K., Yamagata K., Tsuruya K., Yoshida H., Fujimoto S., Asahi K. (2018). Alcohol consumption and incidence of proteinuria: A retrospective cohort study. Clin. Exp. Nephrol..

[11] Li D., Xu J., Liu F., Wang X., Yang H., Li X. (2019). Alcohol drinking and the risk of chronic kidney damage: A meta-analysis of 15 prospective cohort studies. Alcohol Clin. Exp. Res..

[12] Yamamoto R., Nagasawa Y., Iwatani H., Shinzawa M., Obi Y., Teranishi J., Ishigami T., Yamauchi-Takihara K., Nishida M., Rakugi H. (2012). Self-reported sleep duration and prediction of proteinuria: A retrospective cohort study. Am. J. Kidney Dis..

[13] Aoki K., Yamamoto R., Shinzawa M., Kimura Y., Adachi H., Fujii Y., Tomi R., Nakanishi K., Taneike M., Nishida M. (2020). Sleep debt and prevalence of proteinuria in subjects with short sleep duration on weekdays: A cross-sectional study. Clin. Exp. Nephrol..

[14] Fujibayashi K., Fukuda H., Yokokawa H., Haniu T., Oka F., Ooike M., Gunji T., Sasabe N., Okumura M., Iijima K. (2012). Associations between healthy lifestyle behaviors and proteinuria and the estimated glomerular filtration rate (eGFR). J. Atheroscler. Thromb..

[15] Odegaard A.O., Jacobs D.R., Steffen L.M., Van Horn L., Ludwig D.S., Pereira M.A. (2013). Breakfast frequency and development of metabolic risk. Diabetes Care.

[16] Ballon A., Neuenschwander M., Schlesinger S. (2019). Breakfast skipping is associated with increased risk of type 2 diabetes among adults: A systematic review and meta-analysis of prospective cohort studies. J. Nutr..

[17] Chen H., Zhang B., Ge Y., Shi H., Song S., Xue W., Li J., Fu K., Chen X., Teng W. (2020). Association between skipping breakfast and risk of cardiovascular disease and all cause mortality: A meta-analysis. Clin. Nutr..

[18] Kutsuma A., Nakajima K., Suwa K. (2014). Potential association between breakfast skipping and concomitant late-night-dinner eating with metabolic syndrome and proteinuria in the Japanese population. Scientifica.

[19] Kim Y.J., Yoon J.H., Choi H.S., Kim C.S., Bae E.H., Ma S.K., Kim S.W. (2020). Meal frequency and skipping breakfast are associated with chronic kidney disease. Nutrients.

[20] Mekary R.A., Giovannucci E., Willett W.C., van Dam R.M., Hu F.B. (2012). Eating patterns and type 2 diabetes risk in men: Breakfast omission, eating frequency, and snacking. Am. J. Clin. Nutr..

[21] Matsuo S., Imai E., Horio M., Yasuda Y., Tomita K., Nitta K., Yamagata K., Tomino Y., Yokoyama H., Hishida A. (2009). Revised equations for estimated GFR from serum creatinine in Japan. Am. J. Kidney Dis..

[22] Ganda O.P. (2016). Comment on Jakubowicz et al. fasting until noon triggers increased postprandial hyperglycemia and impaired insulin response after lunch and dinner in individuals with type 2 diabetes: A randomized clinical trial. Diabetes Care.

[23] Jakubowicz D., Wainstein J., Ahren B., Landau Z., Bar-Dayan Y., Froy O. (2015). Fasting until noon triggers increased postprandial hyperglycemia and impaired insulin response after lunch and dinner in individuals with type 2 diabetes: A randomized clinical trial. Diabetes Care.

[24] Ogata H., Kayaba M., Tanaka Y., Yajima K., Iwayama K., Ando A., Park I., Kiyono K., Omi N., Satoh M. (2019). Effect of skipping breakfast for 6 days on energy metabolism and diurnal rhythm of blood glucose in young healthy Japanese males. Am. J. Clin. Nutr..

[25] Mah E., Bruno R.S. (2012). Postprandial hyperglycemia on vascular endothelial function: Mechanisms and consequences. Nutr. Res..

[26] Tominaga M., Eguchi H., Manaka H., Igarashi K., Kato T., Sekikawa A. (1999). Impaired glucose tolerance is a risk factor for cardiovascular disease, but not impaired fasting glucose. The Funagata diabetes study. Diabetes Care.

[27] Hu G., Qiao Q., Tuomilehto J. (2001). Glucose tolerance and cardiovascular mortality. Arch. Intern. Med..

[28] Nagata C., Tamura T., Wada K., Konishi K., Goto Y., Nagao Y., Ishihara K., Yamamoto S. (2017). Sleep duration, nightshift work, and the timing of meals and urinary levels of 8-isoprostane and 6-sulfatoxymelatonin in Japanese women. Chronobiol. Int..

[29] Oikonomou E., Lazaros G., Christoforatou E., Chasikidis C., Vavouranaki G., Vogiatzi G., Papamikroulis G.A., Tsalamandris S., Gergiopoulos G., Mazaris S. (2019). Breakfast association with arterial stiffness and carotid atherosclerotic burden. Insights from the ‘Corinthia’ study. Nutr. Metab. Cardiovasc. Dis..

[30] Duni A., Liakopoulos V., Roumeliotis S., Peschos D., Dounousi E. (2019). Oxidative stress in the pathogenesis and evolution of chronic kidney disease: Untangling Ariadne’s thread. Int. J. Mol. Sci..

[31] Fujiwara T., Sato N., Awaji H., Sakamoto H., Nakata R. (2009). Skipping breakfast adversely affects menstrual disorders in young college students. Int. J. Food Sci. Nutr..

[32] Hu Z., Tang L., Chen L., Kaminga A.C., Xu H. (2020). Prevalence and risk factors associated with primary dysmenorrhea among Chinese female university students: A cross-sectional study. J. Pediatr. Adolesc. Gynecol..

[33] Yeh M.L., Chen H.H., So E.C., Liu C.F. (2004). A study of serum malondialdehyde and interleukin-6 levels in young women with dysmenorrhea in Taiwan. Life Sci..

[34] Dikensoy E., Balat O., Pençe S., Balat A., Çekmen M., Yurekli M. (2008). Malondialdehyde, nitric oxide and adrenomedullin levels in patients with primary dysmenorrhea. J. Obstet. Gynaecol. Res..

[35] Jhee J.H., Kee Y.K., Park J.T., Chang T.-I., Kang E.W., Yoo T.-H., Kang S.-W., Han S.H. (2019). A diet rich in vegetables and fruit and Incident CKD: A community-based prospective cohort study. Am. J. Kidney Dis..

[36] Park I., Xun P., Tsinovoi C.L., Klemmer P., Liu K., He K. (2020). Intakes of long-chain omega-3 polyunsaturated fatty acids and non-fried fish in relation to incidence of chronic kidney disease in young adults: A 25-year follow-up. Eur. J. Nutr..

[37] Kant A.K., Graubard B.I. (2015). Within-person comparison of eating behaviors, time of eating, and dietary intake on days with and without breakfast: NHANES 2005-20101-3. Am. J. Clin. Nutr..

[38] Umemura U., Ishimori M., Kobayashi T., Tamura Y., Koike K.A., Shimamoto T., Iso H. (2005). Possible effects of diets on serum lipids, fatty acids and blood pressure levels in male and female Japanese university students. Environ. Health Prev. Med..

[39] Ajjarapu A.S., Hinkle S.N., Li M., Francis E.C., Zhang C. (2019). Dietary patterns and renal health outcomes in the general population: A review focusing on prospective studies. Nutrients.

